# Supplementary material for: Analysis of Correlation Effects of Double Mutations in Enzymes: A Revised Residual-Contact Network Clique Model
Source: Int J Mol Sci. 2024 Aug 22;25(16):9114. doi: 10.3390/ijms25169114 (PMC11354518; doi:10.3390/ijms25169114)
Supplement: Supplementary file 1 [file ijms-25-09114-s001.zip › Supplement Figure.pdf]

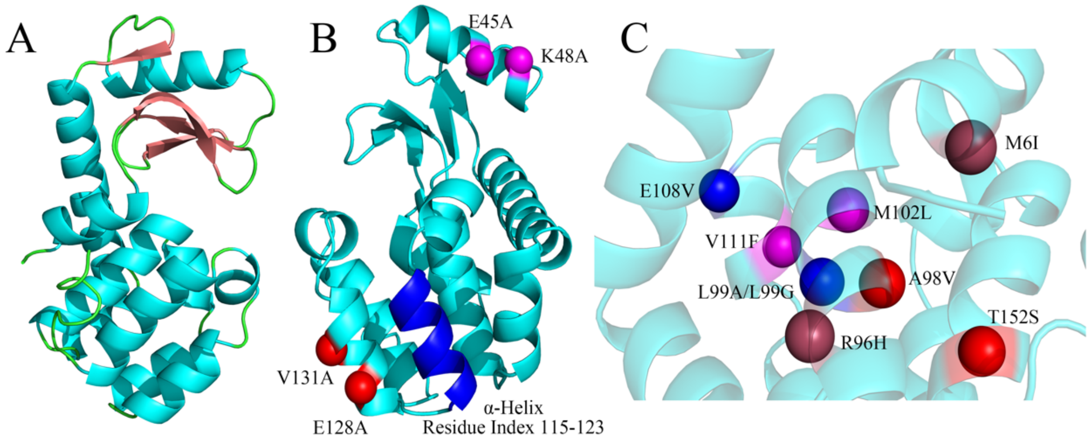


**Figure S1.** (**A**) Protein structure of phage t4 lysozyme; (**B**)Distribution of double mutation data in the same α-helix, Six pairs of these double mutations were distributed in the α-helix with residue index 115-123; (**C**)Distribution of double mutation data in different α-helixes
