# Supplementary material for: Analysis of Correlation Effects of Double Mutations in Enzymes: A Revised Residual-Contact Network Clique Model
Source: Int J Mol Sci. 2024 Aug 22;25(16):9114. doi: 10.3390/ijms25169114 (PMC11354518; doi:10.3390/ijms25169114)
Supplement: Supplementary file 1 [file ijms-25-09114-s001.zip › Supplementary Document.pdf]

Title: Analysis of Correlation Effects of Double Mutations in Enzymes: A Revised Residual-Contact Network Clique Model

Authors: Xianbo Zhang, Junpeng Xu and Dengming Ming *

In the L37A/E75V double mutant, the pattern of forces between L37A/E75V in the double mutant is more similar to that in the E75V single mutant structure, as indicated by the more similar $\Delta\Delta G$ of the mutation. In the single E75V mutation structure (Figure S2A), the hydrogen bond between E75V and H121 disappears, the stable triangular force structure between E75/Y91/H121 is broken, and there is more space between Y91 and H121, and van der Waals interactions appear between Y91 and L37. In contrast, in the L37A single mutant (Figure S2B), the E75/Y91/ H121 stable triangle remains, and L37A is not affected by this system. In the L37A/ E75V double mutant (Figure S2C), the force pattern in this region is more similar to that in the E75V single mutant. Therefore, the double mutant folding free energy is more identical to the E75V single mutant.

In the double mutant of V23F/I72V, the introduction of a benzene ring in the side chain of amino acid 23 resulted in strong van der Waals forces with 62/63/66 in the nearby α-helices (Figure S2D),causing the mutation of I72V, despite the shortening of

the length of the side chain, to produce van der Waals forces with V66 at a more considerable distance from the threshold of the forces due to the more compact internal structure of the sites V23F and V66, and perturbing the originally independent system of the forces, resulting in the occurrence of the mutational non-additivity(Figure S2E-F)

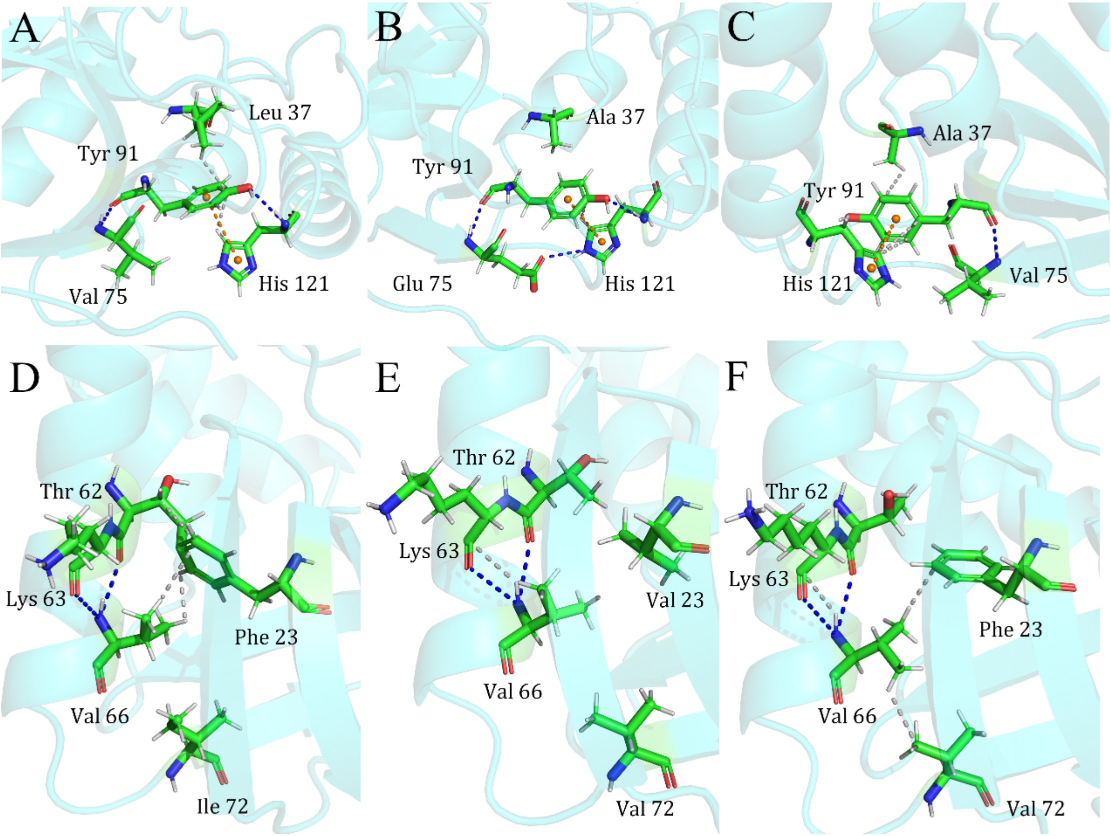

Figure **S2** Staphylococcal nuclease single mutant and double mutant structures in the case of local interaction forces. (A)E75V mutant;(B)L37A mutant; (C) L37A/E75V double mutant; (D) V23F mutant; (E) I72V mutant; (F) V23F/I72V double mutant The grey connections are van der Waals forces, the blue ones are hydrogen bonds, the orange ones are π-π stacks, and the yellow ones are the distances between the atoms in the residues.
